# Asynchronous online learning as a key tool to adapt to new educational needs in radiology during the COVID-19 pandemic

**DOI:** 10.1080/10872981.2022.2118116

**Published:** 2022-09-06

**Authors:** Pau Xiberta, Imma Boada, Santiago Thió-Henestrosa, Salvador Pedraza, Víctor Pineda

**Affiliations:** a Graphics and Imaging Laboratory, Universitat de Girona, Girona, Catalonia; b Departament d’Informàtica, Matemàtica Aplicada i Estadística, Universitat de Girona, Girona, Catalonia; c Centre de Diagnòstic per la Imatge, Hospital Clínic de Barcelona, Barcelona, Catalonia; d Department of Radiology and IDIBGI, Hospital Universitari Dr Josep Trueta (Institut de Diagnòstic per la Imatge), Girona, Catalonia

**Keywords:** Asynchronous online e-learning, COVID-19 pandemic, radiology training, continuing medical education (CME), chest X-ray (CXR)

## Abstract

The risk of contagion and the lockdown caused by the COVID-19 pandemic forced a change in teaching methodologies in radiology. New knowledge about the disease that was being acquired on a daily basis needed to be rapidly spread worldwide, but the restrictions imposed made it difficult to share this information. This paper describes the methodology applied to design and launch a practice-based course on chest X-ray suggestive of COVID-19 right after the pandemic started, and aims to determine whether asynchronous online learning tools for radiology education are useful and acceptable to general practitioners and other medical personnel during a pandemic. The study was carried out from April to October 2020 and involved 2632 participants. Pre- and post-testing was used to assess the participants’ gain of knowledge in the course content (paired t-tests and chi-squared tests of independence). A five-point Likert scale questionnaire inspired by the technological acceptance model (TAM) was provided to evaluate the e-learning methodology (ANOVA tests). The results from the pre- and post-tests showed that there were significant differences in the scores before and after completing the course (sample size = 2632, response rate = 56%, p<0.001). As for the questionnaire, all questions surpassed 4.5 out of 5, including those referring to perceived ease of use and perceived usefulness, and no significant differences were found between experienced and inexperienced participants (sample size = 2535, response rate = 53%, p=0.85). The analysis suggests that the applied methodology is flexible enough to adapt to complex situations, and is useful to improve knowledge on the subject of the course. Furthermore, a wide acceptance of the teaching methodology is confirmed for all technological profiles, pushing for and endorsing a more widespread use of online platforms in the domain of radiology continuing education.

## Introduction

The sudden appearance of the SARS-CoV-2 coronavirus disease of 2019 (COVID-19) entailed a titanic research effort to uncover new findings on symptoms and treatment concerning the disease. This research had to be efficient, implying that new strategies to fight the virus had to be quickly communicated throughout the world as they became known [1]. However, the situation also required being aware that the information was in high demand, and this speed of dissemination resulted in some inadequate content [2,3]. This need for suitable information particularly affected the medical community, where rapid sharing of scientific knowledge was essential [4]. In the field of radiology, information related to COVID-19 was also changing rapidly [5,6], and new protocols needed to be efficiently designed and explained. However, the pandemic situation itself affected the way of interacting and sharing these new data, so that it was necessary to look for other learning alternatives.

Since the establishment of e-learning technologies, other trends have emerged taking advantage of the increasingly intensive use of new technologies. Some examples are m-learning (mobile learning), which could be defined as using mobile technologies to facilitate learning, and u-learning (ubiquitous learning), which is learning anywhere, at any time, and in any way [7,8]. All these paradigms take into account asynchronous learning, in which the student can decide the most appropriate place and time to learn.

In the era of ubiquitous information, medical education is also heading towards the digital world. It will be necessary to educate medical professionals so that they know how to deal with the current technological universe and, above all, how to adapt to new changing situations [9]. Therefore, the learning methods used to educate medical professionals should be reconsidered. Several studies showed that online learning is not worse than traditional learning; thus, with the advantages of online learning in mind, it can be considered as a potential method in medical teaching [10]. This transformation accelerated with the situation generated by COVID-19. To minimise the loss of learning time due to the pandemic, teaching methodologies and content were adapted to remote formats. In order to reduce the risk of contagion, e-learning technologies became even more relevant considering their potential to replace face-to-face classes in educational institutions [11–14]. These changes were experienced in most settings, such as businesses and hospitals, where working methodologies were redefined to adapt to the new situation [15]. In medical education, asynchronous online approaches became essential in allowing teaching and learning to take place anywhere without restrictions [16,17]. While transitioning the entire curriculum to an asynchronous platform does not seem recommended, it does work with supporting content. Consequently, the blended learning approach, i.e., the combination of traditional and online methods, seems to be a promising option for curricular adaptations [18].

Radiology has a unique opportunity to lead this change in medical education. The importance of imaging and the interaction that current technological tools allow make it an ideal discipline for this adaptation. Indeed, the impact on radiology education due to the COVID-19 pandemic resulted in schedule disruptions [19–21], and forced medical schools and health institutions to restructure the curriculum and the teaching methodologies [22,23] by embracing the use of new technologies [24,25] while maintaining the well-being of learners [26]. To reinforce this new instructional methodology, both for residents and general practitioners in continuing education sessions, radiology societies provided free learning material for training [27–31]. However, despite the obvious advantages of these new formats, such as the digital exploration of images, efficiency in terms of time and resources, the removal of physical and temporal barriers, and the possibility of targeting a much larger audience, there are also limitations. These difficulties include a higher workload in preparing courses, issues with motivating students to follow online courses, and a lack of social interaction [32,33]. This digital transformation took for granted that all the actors, i.e., teachers, students and educational centres, had the skills, the knowledge and the infrastructure required to apply online methodologies, although in some cases this was not true [34–36]. Moreover, it was not always possible to allocate the necessary time for digital preparation, and a digital competence was assumed that in some cases required an extra effort.

It is in this context where we want to present the experience of using interactive asynchronous e-learning technologies as a channel to rapidly promote the day-to-day acquired knowledge on COVID-19 radiological diagnosis to general practitioners and other medical personnel. The aim of this paper is to demonstrate the agility that online learning tools provide to adapt to a new situation, designing and launching a continuing medical education course on COVID-19 approximately one month after the start of the pandemic, and also evaluate whether e-learning as a teaching methodology for radiology education is useful and acceptable to general practitioners and other medical personnel during a pandemic.

## Methods

### The radiological education platform (RadEd)

The online course evaluated in this study was developed using the RadEd tool. RadEd [37] is a web-based e-learning platform designed to complement teaching and learning of subjects that require the interaction with radiological images. It is available for PC, tablets and smartphones and it is multilingual. RadEd allows the creation of modules (or courses) grouped by topics which can contain different levels of sections and subsections. These items can contain theory and exercises. There are different types of exercises, such as multiple-choice questions, identification of image regions and image labelling, either by selecting points on the image or by drawing polylines that have to overlap one or more predefined areas. Other more specific exercise types exist, but they are not relevant to this study. All of them can be corrected online using the corresponding correction strategy integrated into the same platform. The platform provides specific editors for teachers to create theory material and exercises, and functionalities to control learners’ work and visualise their progress with respect to other learners in the course, amongst others. For more details, see [37].

### A course on chest radiology suggestive of COVID-19

Although real-time reverse transcription polymerase chain reaction (RT-qPCR) is the gold standard in COVID-19 diagnosis, its large response time became a main limitation, especially in emergency departments where rapid responses were desired [38,39]. To overcome this limitation, one of the initial proposals was to apply chest X-ray (CXR) imaging evaluation in patients with clinical-epidemiological suspicion of COVID-19 to rapidly identify positive cases while waiting for RT-qPCR results [40–42]. Indeed, a large number of hospitals employed CXR as the first-line method to screen COVID-19 cases [43,44]. In our hospital, CXR was used as a first screening strategy during the first wave of the pandemic. It is the reference hospital of the region and had to treat a large number of patients with COVID-19. In this process, different considerations related to the findings, the characteristics of CXR and the evolution of COVID-19 had to be taken into account [45–47].

At the time, our imaging database had many cases to illustrate all the aforementioned considerations. For this reason, we decided to create an online course using our previous experience in e-learning methodologies [48] and e-learning platform development [37]. The course was created to help professionals identify findings suggesting pulmonary involvement by COVID-19 through CXR images, as well as other findings that could point to other pathologies. The course was prepared by a group of radiologists with more than 20 years of experience both diagnosing and teaching. It was organised in six topics and its completion time was estimated at one hour. Each topic had theory and practice (see Table 1). The course was made available through a link to a website [49].

**Table 1.** Topics of the course on chest radiology suggestive of COVID-19.

Topic	Theory pages	Exercises
Presentation	1	3
Introduction to chest X-ray	5	2
Basics on chest X-ray of COVID-19	2	2
Chest X-ray findings of COVID-19	15	12
Indications and radiological monitoring	2	3
Standardised radiology report and score of severity stratification	2	3
Final Quiz	1	3

The description of the main concepts was introduced in the theoretical content of each topic so as to be able to properly identify and interpret the radiological findings based on their radiological appearance and diagnostic value. To consolidate the knowledge of these concepts, exercises with practical cases were carried out in each topic where participants had to identify and interpret the key findings. The first and the last topic corresponded to tests to evaluate the knowledge of the participants before and after taking the course.

In all the topics, the theoretical content was presented as a set of individual pages, each of which always incorporated an interactive image to illustrate the concepts. The exercises were also associated with an image on which learners could interact to better explore the case before answering the question. Both in the theory and in the exercises, the image always played the main role on the page. For each non-assessable exercise, learners had three attempts to solve it. Immediate feedback was provided after submitting an answer, stating whether it was correct or incorrect, and providing a text with more information about the solution when it was correctly solved or when no more attempts were left. Moreover, support from the authors was provided via email.

### Participants

To promote the course and engage the maximum number of participants, it was advertised in the Institute of Diagnostic Imaging (Institut de Diagnòstic per la Imatge, IDI) official website. IDI is a public company attached to Catalan Health department whose aim is the management, administration and execution of diagnostic imaging and nuclear medicine services of Catalan hospitals. Amongst its functions, it develops teaching and research programs, and collaborates with the university and the rest of competent institutions in teaching and research in terms of diagnostic imaging and nuclear medicine, besides fostering innovation and managing knowledge. The course was also announced in different radiology forums.

**Figure 1.**
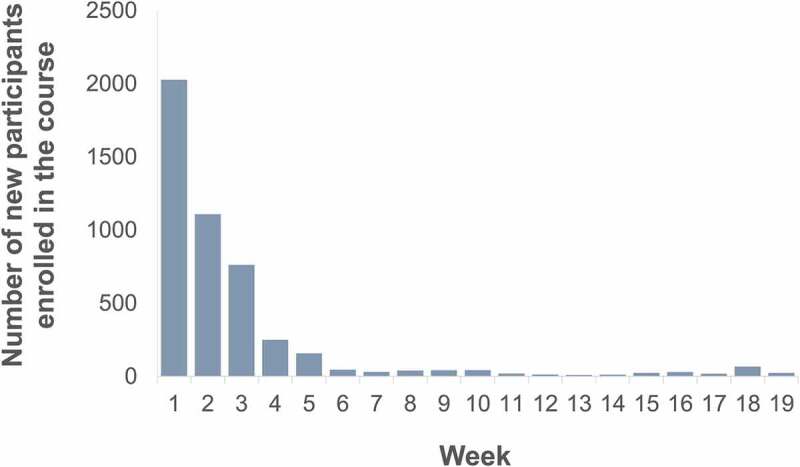
Number of participants enrolled in the course per week.

After the announcement, the response was immediate (see Figure 1), and there are currently more than 5000 participants enrolled in it. During the first week, more than 2000 users were enrolled in the course, and since then the number of registered users decreased, although there was and continues to be interest in the course.

The course was free and to enrol in it a registration process was required. The registration process included two steps: data filling and confirmation. First, users were asked to fill a form with some relevant data, such as the preferred language to perform the course (Catalan, English and Spanish available), the name and the surname, the username to access the platform, and the email that would be used to complete the registration process. Other optional data were the form of address, the occupational category (Physician – Radiologist, Physician – Not Radiologist, and Other Healthcare Providers), the work centre, the country and the city. Once the form was filled, users had to accept the data treatment policy to proceed. If the relevant data were valid, users received a confirmation email to ensure the email address was correct. After the confirmation, users received another email with the access data and some instructions about the course and the platform. Once registered, participants were granted access to the course for two weeks to complete it.

To carry out this study we considered 2632 out of 4739 participants enrolled from April 2020 to October 2020, i.e., those who answered all the exercises of the tests (first and last topic) and completed at least 80% of the course. The experiment in which they participated was conducted according to the Declaration of Helsinki principles. The data treatment policy approval required to enrol in the course received a favourable opinion by the Information Management Technical Committee (CTGI) of the Universitat de Girona. The study is based on anonymous data without allowing the identification of individuals, and participation was completely voluntary.

### Experimental design

The evaluation of the course was carried out taking into account two different approaches: how the course improved the acquired knowledge of the participants, and how this online methodology was evaluated by the participants. Both approaches analysed the results globally, but also by age, professional category and previous experience in online courses.

#### Acquired knowledge evaluation

**Figure 2.**
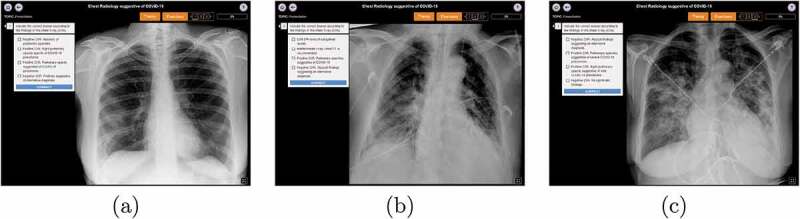
The (a) first, (b) second and (c) third exercise used to perform the pre- and post-test for the evaluation of the acquired knowledge.

Regarding the acquisition of knowledge, it was evaluated by comparing the results of a pre- and a post-test. The first topic of the course served the purpose of evaluating the knowledge of the learners on the subject before the start of the course (the pre-test), without giving them feedback. Once they had been able to learn the concepts and practise, the same exercises from the first topic were presented in the final quiz as a post-test. The three exercises of the pre- and post-test are depicted in Figure 2, each exercise corresponding to a different case.

For each exercise of the pre-test, learners had a single attempt to solve it and did not know the correct result after submitting the answer. For the post-test, learners still had one attempt to solve each exercise, but the platform provided immediate feedback when the answer was submitted. To carry out the evaluation, the exercises were scored 0 if the submitted answer was incorrect, and 1 if it was correct. By comparing how each participant performed in the pre- and the post-test, it could be stated whether this course improved or not their knowledge on the subject.

While it is true that the lack of a control group impoverishes the experimental design, the main interest was to spread knowledge about the diagnosis of COVID-19 as quickly as possible. Spending more time creating an alternative course and excluding a group of participants from this flexible methodology did not seem fair or appropriate. Therefore, the study only evaluates whether knowledge improved, but cannot confirm whether this methodology is better than another.

#### E-learning methodology evaluation

The evaluation of the e-learning methodology was inspired by the technology acceptance model (TAM) [50] and its two main constructs: perceived usefulness (PU), which corresponds to the degree to which a person believes that using a particular system would enhance their job performance, and perceived ease of use (PEOU), which represents the degree to which a person believes that using a particular system would be free from effort. After completing the course (or at least 80% of the exercises), participants were invited to fill out the questionnaire presented in Table 2, where platform-related questions (Q03-Q06) were associated with PEOU, and content-related questions (Q07-Q10) were associated with PU. Moreover, two general questions (Q01-Q02) were included to summarise the user acceptance (UA). To fill out the questionnaire, a Likert scale (1 = total disagreement, to 5 = total agreement) was used [51]. The evaluation took into account 2535 surveys, which correspond to those that were provided by the participants. However, a few surveys lacked some data, so the sample for some analyses may differ.

**Table 2.** Participants’ questionnaire to evaluate the e-learning methodology.

**General Information**
Age
How many online courses have you participated in, aside from this one? [0, 1 to 3, > 3]
Which device have you used preferentially to take the course? [Computer, Tablet, Others]
Do you think that the smartphone version of the platform is useful? [Yes, No]
**Global Evaluation (**1**= Totally disagree, to** 5 **=Totally agree)**
(Q01) Globally, I favourably evaluate the course
(Q02) I would recommend this teaching methodology to my teammates
**Usability (**1** = Totally disagree, to** 5 **= Totally agree)**
(Q03) It was easy for me to interact with images
(Q04) It was easy for me to access and navigate through the content pages
(Q05) It was easy for me to access and navigate through the exercises
(Q06) It was easy for me to identify each icon with its function
**Content (**1** = Totally disagree, to** 5 **= Totally agree)**
(Q07) The topics in which the course was structured are appropriate
(Q08) The course content met my expectations
(Q09) The balance between exercises and theory was appropriate
(Q10) Participating in this activity will allow me to improve elements of my daily work

### Statistical analysis

To analyse the knowledge gain in the course content, the mean of each exercise or the mean of groups of exercises of the pre- and post-tests were compared by means of a paired t-test. To evaluate the result of the post-test by age, professional category and experience in online courses, a chi-squared test of independence was used.

Regarding the evaluation of the quality of the course, the questions were grouped according to the three main categories of the questionnaire: global evaluation, usability and content. The questions of each category were added and divided by the number of questions in order to have the same scale. To compare the means by age, professional category and experience in online courses, an ANOVA test was used (Welch’s ANOVA if unequal variances were observed).

## Results

### Participants’ profile

**Figure 3.**
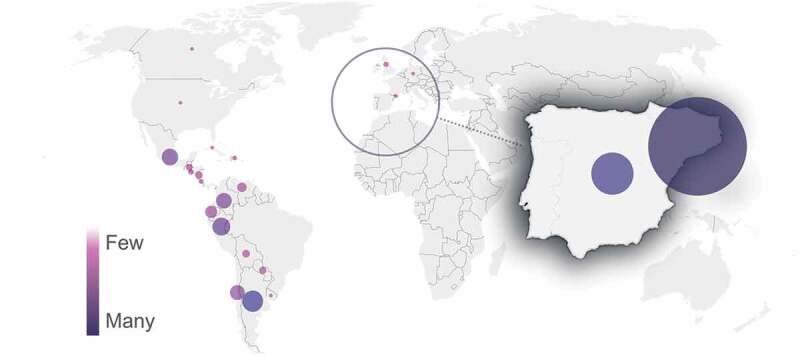
Distribution of participants by country of origin (the area of the bubble is proportional to the number of participants).

**Figure 4.**
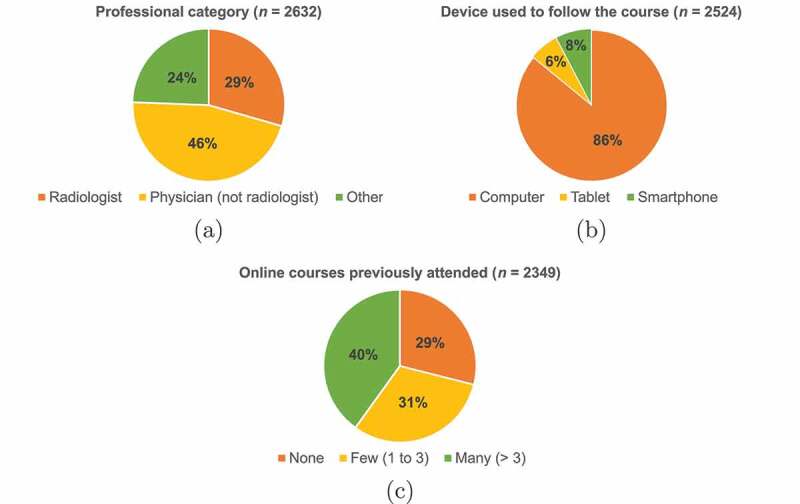
Distribution of participants by (a) professional category, (b) device used to follow the course, and (c) number of online courses previously attended. For all charts, n represents the number of participants who provided the corresponding information in the course registration form or in the evaluation questionnaire.

The participants’ profile included radiologists, physicians (not radiologists), and other healthcare providers which include medical students, residents and technical radiologists. Even though the course could be taken in Catalan, English or Spanish in order to spread this knowledge globally, given the urgency of the situation, most of the participants were from Catalonia (see Figure 3). This distribution was already expected, as the course was not promoted through many international radiology associations. This and other profile-related information was obtained from the data collected both in the course registration process and in the course evaluation survey. These data comprised questions about professional category, age, gender, region, device used to follow the course, and number of online courses previously attended. The number of participants within each of these categories is summarised in Table 3, and the distribution of participants by some of the characteristics is depicted in Figure 4. Of those who provided such information, it is possible to describe the average participant of the course as a woman between 30 and 50 years old, physician but not radiologist, from Catalonia, who used a computer to follow the course, and with experience in online courses.

**Table 3.** Number (n) and percentage (%) of participants within each of the analysed categories (only those who provided the information are considered).

		n	%
Professional category	Physician – Radiologist	775	29.45%
	Physician – Not Radiologist	1215	**46.16%**
	Other Healthcare Providers	642	24.39%
Age	< 24	81	4.64%
	24 to 29	381	21.83%
	30 to 50	950	**54.44%**
	> 50	333	19.08%
Gender	Female	1324	**67.93%**
	Male	625	32.07%
Region	Catalonia	1923	**73.62%**
	Spain	339	12.98%
	Other	350	13.40%
Device used to follow the course	Computer	2169	**85.94%**
	Smartphone	194	7.69%
	Tablet	161	6.38%
Online courses previously attended	None	680	28.95%
	Few (1 to 3)	728	30.99%
	Many (> 3)	941	**40.06%**

### Pre- and post-testing results

To evaluate the acquired knowledge during the course, the results of the three exercises of the pre- and post-test were analysed. First, each exercise was considered individually, while in a second analysis they were considered as a whole.

A summary of the results of the independently evaluated pre- and post-test exercises is shown in Table 4. In all exercises there was a significant difference in the mean before and after performing the course, i.e., between the pre-test and the post-test. *Exercise 1* improved the mean of the answers by 0.17 points (from 0.53 to 0.70), and *Exercise 2* improved it by 0.10 points (from 0.81 to 0.91). On the contrary, *Exercise 3* remained similar after taking the course, worsening the mean by 0.02 points (from 0.09 to 0.07).

**Table 4.** Comparison of independently evaluated pre- and post-test exercises.

		mean	sd^a^	n^b^	p-value
*Exercise 1*	Pre-test	0.53	0.50	2632	**< 0.001**
	Post-test	0.70	0.46	2632	
*Exercise 2*	Pre-test	0.81	0.39	2632	**< 0.001**
	Post-test	0.91	0.29	2632	
*Exercise 3*	Pre-test	0.09	0.29	2632	**< 0.001**
	Post-test	0.07	0.26	2632	

a Standard deviation

b Number of participants

As for the second analysis, a summary of the results of the pre- and post-test exercises evaluated as a whole is shown in Table 5. Similarly, when all the exercises were considered together, there was also a significant difference in the mean between the pre- and post-test. In this case, the mean improved by 0.24 points (from 1.44 to 1.68).

**Table 5.** Comparison of pre- and post-test exercises considered as a whole.

		mean	sd^a^	n^b^	p-value
All exercises	Pre-test	1.44	0.71	2632	**< 0.001**
	Post-test	1.68	0.61	2632	

a Standard deviation

b Number of participants

Apart from analysing the global differences between the results of the pre- and post-test, we also evaluated the scores of the same exercises of the post-test taking into account some characteristics of the participants’ profile. Thus, Table 6 presents the scores of these exercises analysed by age. There were no significant differences except for *Exercise 1*, for which the youngest participants (under 24 years old) had a lower proportion of correct answers.

**Table 6.** Scores of the post-test exercises by age.

	Post-test *Exercise 1*		Post-test *Exercise 2*		Post-test *Exercise 3*	
Age	Correct	Incorrect	Correct	Incorrect	Correct	Incorrect
< 24	47	34	71	10	3	78
	(58%)	(42%)	(87.7%)	(12.3%)	(3.7%)	(96.3%)
24 to 29	269	112	349	32	22	359
	(70.6%)	(29.4%)	(91.6%)	(8.4%)	(5.8%)	(94.2%)
30 to 50	708	242	876	74	74	876
	(74.5%)	(25.5%)	(92.2%)	(7.8%)	(7.8%)	(92.2%)
> 50	225	108	294	39	25	308
	(67.6%)	(32.4%)	(88.3%)	(11.7%)	(7.5%)	(92.5%)
p-value	**0.003**		**0.111**		**0.362**	

When the analysis of the post-test scores was carried out considering the professional category, as shown in Table 7, significant differences were found in *Exercise 1* and *Exercise 3*. Regarding *Exercise 1*, those participants who were not physicians (category named *Other Healthcare Providers*) had a lower proportion of correct answers, while in *Exercise 3* radiologists were the group who performed better.

**Table 7.** Scores of the post-test exercises by professional category.

	Post-test *Exercise 1*		Post-test *Exercise 2*		Post-test *Exercise 3*	
Professional category	Correct	Incorrect	Correct	Incorrect	Correct	Incorrect
Physician – Radiologist	598	177	693	82	103	672
	(77.2%)	(22.8%)	(89.4%)	(10.6%)	(13.3%)	(86.7%)
Physician – Not Radiologist	884	331	1119	96	51	1164
	(72.8%)	(27.2%)	(92.1%)	(7.9%)	(4.2%)	(95.8%)
Other Healthcare Providers	361	281	580	62	32	610
	(56.2%)	(43.8%)	(90.3%)	(9.7%)	(5%)	(95%)
p-value	**< 0.001**		**0.111**		**< 0.001**	

Finally, Table 8 shows the results of the post-test scores analysed by the participants’ experience in online courses. In this case, no significant differences were found.

**Table 8.** Scores of the post-test exercises by number of online courses previously attended.

	Post-test *Exercise 1*		Post-test *Exercise 2*		Post-test *Exercise 3*	
Number of online courses previously attended	Correct	Incorrect	Correct	Incorrect	Correct	Incorrect
None	465	215	617	63	51	629
	(68.4%)	(31.6%)	(90.7%)	(9.3%)	(7.5%)	(92.5%)
Few (1 to 3)	517	211	658	70	61	667
	(71%)	(29%)	(90.4%)	(9.6%)	(8.4%)	(91.6%)
Many (> 3)	667	274	861	80	54	887
	(70.9%)	(29.1%)	(91.5%)	(8.5%)	(5.7%)	(94.3%)
p-value	**0.469**		**0.718**		**0.099**	

### E-learning methodology evaluation results

The evaluation of the e-learning methodology was carried out using the data provided by the participants in the questionnaire presented in Table 2. This questionnaire was divided into three categories: global evaluation (UA), usability (PEOU) and content (PU). For each category, only those participants who provided the corresponding information were analysed.

Thus, according to the results, the quality of the course and the e-learning methodology was excellent, since the mean was higher than 4.5 out of 5 in all three categories. Furthermore, there was a very low deviation (coefficient of variation lower than 0.14), a fact that indicates a high homogeneity in the answers. A summary of the responses provided by the participants is presented in Table 9.

**Table 9.** Summary of the responses of the e-learning methodology evaluation questionnaire.

Category	mean	sd^a^	n^b^
Global evaluation	4.62	0.60	2484
Usability	4.57	0.57	2470
Content	4.53	0.63	2460

a Standard deviation

b Number of participants

Similar to the analysis performed for the results of the pre- and post-test, the responses of the questionnaire were also evaluated taking into account the same characteristics of the participants’ profile. Accordingly, the results of the evaluation analysed by age are presented in Table 10. There were only significant differences in the global evaluation, where the young participants (under 30 years old) scored a little lower.

**Table 10.** E-learning methodology evaluation by age.

	Global evaluation			Usability			Content		
Age	mean	sd^a^	n^b^	mean	sd^a^	n^b^	mean	sd^a^	n^b^
< 24	4.58	0.61	78	4.52	0.52	80	4.48	0.61	81
24 to 29	4.57	0.64	379	4.59	0.56	376	4.53	0.62	373
30 to 50	4.66	0.53	938	4.62	0.50	936	4.57	0.59	928
> 50	4.70	0.53	329	4.57	0.56	330	4.59	0.59	328
p-value	**0.002**			**0.180**			**0.298**		

a Standard deviation

b Number of participants

The opposite behaviour was obtained when the responses were analysed by professional category, since significant differences were found in the usability and content categories. As shown in Table 11, radiologists scored slightly lower than the other participants.

**Table 11.** E-learning methodology evaluation by professional category.

	Global evaluation			Usability			Content		
Professional category	mean	sd^a^	n^b^	mean	sd^a^	n^b^	mean	sd^a^	n^b^
Physician – Radiologist	4.57	0.64	610	4.51	0.62	674	4.46	0.69	600
Physician – Not Radiologist	4.63	0.58	1152	4.58	0.54	716	4.56	0.61	1149
Other Healthcare Providers	4.65	0.59	722	4.57	0.56	933	4.55	0.63	711
p-value	**0.058**			**0.022**			**0.011**		

a Standard deviation

b Number of participants

Finally, when the analysis of the e-learning methodology evaluation was carried out considering the participants’ previous experience in online courses, no significant differences were found, as revealed in Table 12.

**Table 12.** E-learning methodology evaluation by number of online courses previously attended.

	Global evaluation			Usability			Content		
Number of online courses previously attended	mean	sd^a^	n^b^	mean	sd^a^	n^b^	mean	sd^a^	n^b^
None	4.61	0.63	673	4.55	0.61	606	4.52	0.67	661
Few (1 to 3)	4.63	0.58	723	4.59	0.55	1142	4.54	0.60	718
Many (> 3)	4.62	0.59	932	4.60	0.55	722	4.54	0.63	927
p-value	**0.852**			**0.558**			**0.740**		

a Standard deviation

b Number of participants

## Discussion

The education system changed with COVID-19. Before the pandemic, a large number of educational centres used online resources and e-learning platforms to complement face-to-face instruction. The different teaching models differed, amongst others, in the final contribution of online methodologies to the whole content of the courses. With COVID-19, a need for transforming teaching methodologies to fully online ones was imposed. This transformation occurred at all educational levels, and also in the context of radiology, where there was also the need for transmitting the new knowledge on diagnostic imaging of the disease immediately and throughout the world. In this paper, we presented an online asynchronous learning module that was able to be rapidly deployed at the beginning of the pandemic and was highly valued by learners. The aim of the study was to confirm that such methodologies are flexible enough to adapt to new situations, that they are capable of contributing to the improvement of knowledge, and that they are accepted by participants.

By analysing the scores of the pre- and post-tests as a whole, a statistically significant improvement was detected in the participants’ knowledge, increasing the average score from a failing grade (1.44 out of 3.00, i.e., less than half of the total score) to a passing grade (1.68 out of 3.00). These results are not different from those obtained in other similar studies, where significant differences between the scores were also found and e-learning was determined to be effective during the COVID-19 pandemic [10,52,53]. The proposed e-learning methodology is then useful to improve the knowledge on the subject studied, thus reaching the same conclusion.

However, in the analysis of each individual exercise, an improvement was only detected in the first and second exercises, but not in the third one. We further examined this third exercise, and we realised that it was considerably more difficult than the other ones. We were aware of this difficulty before the start of the course, since adding a more complex case was part of the methodology to evaluate knowledge improvements; nonetheless, correctly solving this exercise responded more to the ability to demonstrate that an expert level was reached rather than to assess whether knowledge was improved, which was actually the ultimate goal. Unlike the first two exercises, the correct answer for *Exercise 3* corresponded to an alternative diagnosis to COVID-19 (specifically, to pulmonary oedema, suggested by the presence of bilateral perihilar alveolar densities with mild left pleural effusion). Although the radiological findings that suggest an alternative diagnosis to COVID-19 were explicitly addressed in the fourth topic of the course, having a background in radiological knowledge may be important to better acquire these concepts. Indeed, radiologists were the group that performed best in this exercise, since their level of expertise in the topic was higher. A similar behaviour occurred in the results of the first exercise, where participants who were physicians scored better than those who were not, and the youngest participants (under 24 years old) scored worse, probably due to the fact that they had not yet completed their academic training. On the other hand, when the scores were analysed by previous experience in online courses, no differences were found, meaning that the e-learning methodology was not a hindrance for the participants to respond as they wanted.

Regarding the evaluation of the e-learning methodology by means of a questionnaire, the obtained results were very satisfactory and highly consistent, with an average score higher than 4.5 out of 5 in all the evaluated dimensions (global evaluation as UA, usability of the platform as PEOU, and course content as PU). These results are in accordance with other studies where online methodologies were positively perceived, feasible, and met the demand of the participants during the pandemic [54–56], recommending above all a blended learning approach, i.e., combining face-to-face and online educational formats. More studies in the field of continuing medical education move along the same lines, emphasising the fact that e-learning was very important amid the pandemic, yet it will not replace traditional forms of medical education, but rather will complement them [57,58]. Nevertheless, mainly due to issues with the technological infrastructure and the lack of interactivity, it is also true that other experiences were not as satisfactory as expected and their participants preferred the traditional method [59,60]. To ensure success, technology requirements must be minimised and courses must be designed with a high level of interaction [61], as the intention of the participants, as well as the usefulness of the technology, are major factors that contribute to successful transitions to online learning [62].

The study presented also analysed the responses to the questionnaire by categories of participants. Despite the fact that younger participants (under 30 years old, including most of the residents) scored slightly lower in the global evaluation, possibly because the exercises were somewhat harder for them, and that radiologists also scored lower to some extent in the usability and content categories, as they may be reasonably more demanding, in the analysis there was a special interest in the participants with no experience in online courses. The results showed that there were no significant differences between experienced and inexperienced participants, thus indicating that the e-learning methodology is suitable for both and that it is able to adapt to any technological profile.

### Limitations

The current study has two main limitations. First, the lack of a control group prevented the proposed online methodology from being compared with a traditional learning approach. The rush to spread the content of the course as a result of the exceptional situation caused by COVID-19 discouraged spending time with an alternative course and isolating a group of participants. The results obtained, therefore, can only compare the performance of the participants before and after taking the course.

Second, the third exercise of the tests evaluated was not very successful. While it is true that a more difficult exercise was included on purpose, it was not taken into account that this strategy could hinder the analysis of knowledge improvement. If the course were to be redesigned, this exercise could remain as long as the course content was widened to better understand the problem.

## Conclusion

Asynchronous online learning is a flexible tool to adapt to complex situations such as that caused by COVID-19. Besides, the use of this methodology to rapidly disseminate changing information regarding imaging and treatment of COVID-19 patients is highly accepted by participants and, although more research is needed, it is effective in improving knowledge acquisition. If the e-learning platform used has the minimum technological requirements and is interactive enough to engage students, then neither the performance nor the assessment of the methodology differs with respect to the technological profile of the participants.

Inexperienced participants were immersed in a digital adventure forced by the impossibility of attending face-to-face classes. Their experience was satisfactory and this should lead to a better acceptance of digital platforms. This is a very promising result that reinforces the idea that online learning should play a more prominent role in continuing education courses. COVID-19 has accelerated changes in the educational system, and the domain of continuing education has been no exception.
